# Association of maternal folate status in the second trimester of pregnancy with the risk of gestational diabetes mellitus

**DOI:** 10.1002/fsn3.1235

**Published:** 2019-10-18

**Authors:** Kaipeng Xie, Pengfei Xu, Ziyi Fu, Xiaohong Gu, Hui Li, Xianwei Cui, Lianghui You, Lijun Zhu, Chenbo Ji, Xirong Guo

**Affiliations:** ^1^ Women's Hospital of Nanjing Medical University The Affiliated Obstetrics and Gynecology Hospital of Nanjing Medical University Nanjing Maternity and Child Health Care Hospital Nanjing China; ^2^ Tongren Hospital Shanghai Jiao Tong University School of Medicine Shanghai China

**Keywords:** gestational diabetes mellitus, red blood cell folate, risk

## Abstract

Interest in the high folate status of pregnant women has increased due to its role in the prevention of neural tube defects (NTDs). The effect of increased red blood cell (RBC) folate status during the second trimester of pregnancy on gestational diabetes mellitus (GDM) remains unclear. We measured RBC folate concentrations by competitive protein‐binding assay and obtained clinical information from electronic medical records. Logistic regression analysis was used to explore the associations of RBC folate concentrations with risks of gestational diabetes mellitus (GDM). We further assessed the potential nonlinear relations between continuous log‐transformed RBC folate concentrations and GDM risk by using the restricted cubic splines. We observed high RBC folate concentrations in GDM patients compared to control group [median (interquartile range, IQR), GDM vs. controls: 1,554.03 (1,240.54–1,949.99) vs. 1,478.83 (1,124.60–1,865.71) nmol/L, *p* = .001]. Notably, high folate concentrations were significantly associated with an increased risk of GDM [RR per 1‐*SD* increase: 1.16 (1.03, 1.30), *p* = .012] after adjustment for maternal age, parity, and body mass index (BMI) at enrollment. In the restricted cubic spline model, a test of the null hypothesis of the linear relationship was rejected (*p* = .001). Our study firstly showed that maternal RBC folate concentrations during the second trimester of pregnancy increase the risk of GDM in a Chinese population. Further randomized clinical trials (RCTs) are warranted to confirm the adverse effect.

## 1 INTRODUCTION

Gestational diabetes mellitus (GDM) has become an emerging global epidemic, affecting 12.9% of pregnant women in the world (Zhu & Zhang, 2016). For pregnant women, GDM is related to adverse pregnancy outcomes and type 2 diabetes in the postpartum period (Farrar et al., 2016). Their offspring is susceptible to develop child obesity and diabetes in adulthood (Bider‐Canfield et al., 2017; Page et al., 2019; Tam et al., 2017). Thus, the identification of potentially modifiable risk factors for GDM is a promising strategy to improve the health of pregnant women and their children.

It has been reported that dietary components are linked to the development of GDM, suggesting that diet intervention may be an ideal strategy for preventing GDM (Agha‐Jaffar, Oliver, Johnston, & Robinson, 2016). Folate aids in the one‐carbon unit transfer for DNA methylation and synthesis of purine and thymidine nucleotides, which is essential for cell division and fetal growth during pregnancy (Ly, Hoyt, Crowell, & Kim, 2012). In past decades, many countries have recommended that women of childbearing age take folic acid (FA) supplementation to prevent neural tube defects (NTDs) (Williams et al., 2015), resulting in an increased folate status in pregnant women (Patel & Sobczynska‐Malefora, 2017). Meanwhile, the concern has been raised as to whether the increased folate status has potential effects on GDM. Zhu et al. (2016) reported that daily FA supplement consumption in early pregnancy was related to the increased risk of GDM. However, the association of serum folate in the third trimester of pregnancy with GDM risk was not observed in a UK population (Sukumar et al., 2016). Obviously, different assessments of folate status (FA supplements and serum folate concentrations), study designs, and sample size may contribute to the conflicting results. Compared to other assessments, RBC folate is more accurate and represents the long‐term folate intake, as RBCs accumulate folate during erythropoiesis only (Shane, 2011). However, no study has been undertaken to systematically evaluate the association of RBC folate concentrations with GDM risk.

In recent years, the World Health Organization (WHO) has summarized historical information about RBC folate concentrations and provided the reference for folate status in the population (Cordero, Crider, Rogers, Cannon, & Berry, 2015). It has defined that RBC folate concentrations <340 nmol/L were considered as folate deficiency, and RBC folate concentrations ≥906 nmol/L were optimal for the prevention of NTDs. However, there is no consensus on the high folate cutoff value. In a classic study by Daly, Kirke, Molloy, Weir, and Scott (1995), RBC folate concentrations ≥1,292 nmol/L showed no risk reduction with regard to NTDs, which may be the potential high folate cutoff value. Other studies tend to use quantiles to examine the effects of high RBC folate concentrations (Colapinto, O'Connor, Sampson, Williams, & Tremblay, 2016). Notably, most of the studies were performed in Western settings. Given different racial/ethnic backgrounds, it is imperative to assess whether the proposed RBC folate values have public health implications in a Chinese population.

In the current study, we describe RBC folate concentrations during the second trimester of pregnancy in a Chinese population and to examine the associations between RBC folate concentrations and the subsequent development of adverse pregnancy outcomes.

## 2 MATERIALS AND METHODS

### 2.1 Study population

A total of 3,195 pregnant women at 19–24 weeks (second trimester of pregnancy) who underwent blood testing for pregnancy complications screening at the Affiliated Obstetrics and Gynecology Hospital of Nanjing Medical University were recruited between May 2013 and May 2014. All the pregnant women provided written informed consent and donated 5 ml peripheral venous blood for further experiments. At enrollment, data on maternal characteristics, including age, weight, height, gestational week, parity (nulliparous or parous), and past medical history were collected by the electronic medical record. Body mass index (BMI) was calculated as weight (kg)/height (m)^2^. At the end of gestation, pregnancy outcomes and delivery information were retrieved from the hospital delivery record according to the criteria of previous studies (Zhou et al., 2014). We excluded pregnant women with chronic disease before pregnancy, multiple gestation, or major congenital fetal anomalies or women whose infants were not delivered in the hospital. For this study, a total of 392 pregnant women with gestational diabetes mellitus (GDM, fasting glucose ≥ 5.5 mmol/L or a 2‐hr plasma glucose ≥ 8.0 mmol/L following a 75‐g oral glucose tolerance test at 24–28 weeks of gestation (Hoffman, Nolan, Wilson, Oats, & Simmons, 1998)) were diagnosed in this cohort. Controls were 1,890 pregnant women without any pregnancy complications or adverse pregnancy outcomes, including GDM, gestational hypertension, preeclampsia, oligohydramnios, and polyhydramnios, or their infants with premature birth, low birthweight, and macrosomia. This study was approved by the Institutional Review Board of the Affiliated Obstetrics and Gynecology Hospital of Nanjing Medical University.

### 2.2 RBC folate concentrations

Maternal whole blood samples were collected at the time of recruitment and used to measure the RBC folate concentrations as previously described (Xie et al., 2018). In brief, blood cells were lysed and RBC folate concentrations were quantified by the Beckman Coulter UniCel DxI 800 Access Immunoassay System. RBC folate concentrations were adjusted for the hematocrit as described by the manufacturer (Shen et al., 2016).

### 2.3 Statistical analysis

For continuous variables, normally distributed data are described as mean ± standard deviation (*SD*), and non‐normal distribution data are presented as median (interquartile range, IQR). Categorical variables are reported as frequency and percentage. Maternal characteristics and RBC folate concentrations were compared between GDM patients and control women using Student's *t* test or Mann–Whitney test for continuous variables. Chi‐square or Fisher's exact test was used for categorical variables. Logistic regressions were performed to determine the odds ratios (ORs) and 95% confidence intervals (CIs) of GDM according to quintiles in the control group and proposed cutoff values of RBC folate concentrations and per 1‐*SD* RBC folate concentrations (log‐transformed variable). The adjusted potential confounders included maternal age, parity, and BMI at enrollment. To determine potential nonlinear relations, we assessed continuous log‐transformed RBC folate concentrations in relation to the risk of GDM by using the restricted cubic splines (Desquilbet & Mariotti, 2010). Nonlinearity was examined by the Wald test. A *p*‐value < .05 was considered to be statistical significance, and all analyses were performed with R software (version 3.2.5).

## 3 RESULTS

Maternal characteristics and RBC folate concentrations of 2,282 pregnant women (392 GDM patients and 1,890 controls) are shown in Table 1. There was a significant difference in age between the case and control groups (mean ± *SD*, 29.01 ± 3.15 vs. 27.89 ± 3.18, *p* < .001). The cases have higher BMI at enrollment in comparison with the control group (mean ± *SD*, 24.33 ± 3.10 vs. 23.16 ± 2.72, *p* < .001). These results indicated that age and BMI at enrollment may be potentially influence factors for GDM. There was no significant difference in parity between the case and control groups (*p* = .422). Notably, the median RBC folate levels in cases group were significantly higher than those in controls (median [IQR], 1,554.03 [1,240.54–1,949.99] vs. 1,478.83 [1,124.60–1,865.71], *p* = .001). Concomitantly, when pregnant women were categorized into five groups according to the quintiles of RBC folate concentrations in the control group or into three groups according to the proposed cutoff values, the distribution of the proportions of RBC folate concentrations was different between case and control groups (all *p* < .001).

**Table 1:** Maternal characteristics and RBC folate concentrations in GDM and control groups

Maternal characteristics	GDM *N* = 392	Control *N* = 1,890	*p*
Age (years)	29.01 ± 3.15	27.89 ± 3.18	<.001
<30	230 (58.67)	1,353 (71.59)	<.001
≥30	162 (41.33)	537 (28.41)	
BMI (kg/m^2^)^a^	24.33 ± 3.10	23.16 ± 2.72	<.001
<25	245 (62.50)	1,474 (77.99)	
≥25	145 (36.99)	398 (21.06)	
Parity			
Nulliparous	366 (93.37)	1,787 (94.55)	.4221
Parous	26 (6.63)	103 (5.45)	
RBC folate (nmol/L)^b^	1,554.03 (1,240.54–1,949.99)	1,478.83 (1,124.60–1,865.71)	1.212 × 10^–3^
RBC folate			
Q1 (≤1,053.46)	36 (9.18)	378 (20.00)	<.001
Q2 (1,053.46–1,311.47)	83 (21.17)	378 (20.00)	
Q3 (1,311.47–1,632.50)	103 (26.28)	378 (20.00)	
Q4 (1,632.50–1,962.06)	75 (19.13)	378 (20.00)	
Q5 (1,962.06–5,108.11)	95 (24.24)	378 (20.00)	
Proposed cut off			
<906 nmol/L	14 (3.57)	173 (9.15)	<.001
906–1,292 nmol/L	96 (24.49)	554 (29.31)	
≥1,292 nmol/L	282 (71.94)	1,163 (61.53)	

Note

Values are expressed as means ± standard deviations, or numbers with percentages (%).

Abbreviation: GDM, gestational diabetes mellitus.

a BMI information was not available in 20 participants, including 2 GDM patients and 18 controls.

b Values are expressed as median (interquartile range, IQR).

Then, we used logistic regression analyses to explore the associations between RBC folate concentrations and GDM (Table 2). Compared to the lowest quintile (Q1), women with high RBC folate concentrations had an increased risk of GDM (Q2: OR = 2.25, 95% CI = 1.47–3.43, *p* = 1.76 × 10^–4^; Q3: OR = 2.65, 95% CI = 1.75–4.02, *p* = 3.88 × 10^–6^; Q4: OR = 1.78, 95% CI = 1.16–2.75, *p* = 8.85 × 10^–3^; Q5: OR = 2.31, 95% CI = 1.51–3.52, *p* = 9.98 × 10^–5^; *P*_trend_ = 0.012) after adjustment for maternal age, parity, and BMI at enrollment. Using the proposed cutoff values, women with RBC folate concentrations from 906 to 1,292 nmol/L and ≥1,292 nmol/L had an increased risk of GDM (OR = 2.17, 95% CI = 1.20–3.95, *p* = .011; OR = 2.76, 95% CI = 1.56–4.89, *p* < .001; *P*_trend_ < 0.001). Consistently, the positive association was still significant when RBC folate concentrations were log‐transformed (RR per 1‐*SD* increase: 1.16, 95% CI = 1.03–1.30, *p* = .012).

**Table 2:** Association of maternal RBC folate concentrations with GDM risk

Groups	GDM OR (95% CI)	
	Univariate	Multivariate
Quintiles		
Q1 (≤1,053.46)	1.00 (ref)	1.00 (ref)
Q2 (1,053.46–1,311.47)	2.31 (1.52, 3.50)	2.25 (1.47, 3.43)
Q3 (1,311.47–1,632.50)	2.86 (1.91, 4.29)	2.65 (1.75, 4.02)
Q4 (1,632.50–1,962.06)	2.08 (1.37, 3.18)	1.78 (1.16, 2.75)
Q5 (1,962.06–5,108.11)	2.64 (1.75, 3.97)	2.31 (1.51, 3.52)
*P*_trend_	3.12 × 10^–4^	0.012
Proposed cutoff (nmol/L)		
<906	1.00 (ref)	1.00 (ref)
906–1,292	2.14 (1.19, 3.85)	2.17 (1.20, 3.95)
≥1,292	3.00 (1.71, 5.25)	2.76 (1.56, 4.89)
*P*_trend_	<0.001	<0.001
Per 1‐*SD* increase^a^	1.22 (1.09, 1.37)	1.16 (1.03, 1.30)

Note

All values are ORs (95% CIs) and determined by using logistic regression.

Adjusted values were adjusted for maternal age, parity, and BMI at enrollment.

a ORs and 95% CIs were per 1‐*SD* increase of log‐transformed RBC folate concentrations.

In the restricted cubic spline model, a test of the null hypothesis of the linear relationship was rejected (*p* = .001), and a significant relationship was observed for high RBC folate concentrations with an increased risk of GDM (*p* < .001). Furthermore, the association of RBC folate concentrations with the risk of GDM was explored after stratifying by maternal age, BMI at enrollment, parity, and birth gender (Table 3). Similar significant associations were observed in pregnant women younger than 30, women with a BMI <25 kg/m^2^, and nulliparous women (all *P*_trend_ < 0.05).

**Table 3:** Stratification analysis between maternal RBC folate concentrations and risk of GDM

Characteristics	Quartile groups of maternal red blood cell folate concentrations					*P*_trend_	Per 1‐*SD* increase
	Q1	Q2	Q3	Q4	Q5		
Maternal age (years)							
<30	1.00 (ref)	2.58 (1.50, 4.44)	3.93 (2.33, 6.66)	2.35 (1.34, 4.11)	3.48 (2.03, 5.94)	1.31 × 10^–4^	1.34 (1.16, 1.55)
≥30	1.00 (ref)	1.91 (0.95, 3.87)	1.48 (0.74, 2.92)	1.20 (0.60, 2.40)	1.38 (0.70, 2.74)	0.827	0.98 (0.80, 1.18)
BMI at enrollment (kg/m^2^)							
<25	1.00 (ref)	2.53 (1.48, 4.30)	2.74 (1.63, 4.62)	2.23 (1.29, 3.83)	2.31 (1.35, 3.95)	0.039	1.11 (1.01, 1.23)
≥25	1.00 (ref)	1.68 (0.83, 3.41)	2.29 (1.15, 4.58)	1.14 (0.55, 2.34)	2.09 (1.05, 4.18)	0.218	1.09 (0.95, 1.26)
Parity							
Nulliparous	1.00 (ref)	2.12 (1.37, 3.29)	2.59 (1.69, 3.98)	1.57 (1.00, 2.46)	2.21 (1.44, 3.41)	0.031	1.10 (1.01, 1.20)
Parous	1.00 (ref)	4.15 (0.77, 22.24)	3.22 (0.61, 17.16)	9.3 (1.64, 52.84)	2.69 (0.39,18.46)	0.145	1.28 (0.92, 1.79)
Birth gender							
Female	1.00 (ref)	1.50 (0.85, 2.64)	2.40 (1.40, 4.09)	1.52 (0.87, 2.67)	2.15 (1.24, 3.75)	0.021	1.14 (1.02, 1.28)
Male	1.00 (ref)	3.56 (1.85, 6.86)	3.06 (1.58, 5.93)	2.18 (1.09, 4.35)	2.60 (1.34,5.03)	0.210	1.08 (0.96,1.22)

Note

Values are expressed as numbers with percentages (%).

All values are ORs (95% CIs) and adjusted for maternal age, BMI at enrollment, gestational weeks at enrollment, gestational weeks at delivery, and infant gender (excluded the stratified factor in each stratum)

## 4 DISCUSSION

This is the first study to assess maternal RBC folate concentrations during the second trimester of pregnancy in a Chinese population and to examine the association with GDM risk. Our results showed that maternal higher folate status is associated with an increased risk of GDM.

**Figure 1:**
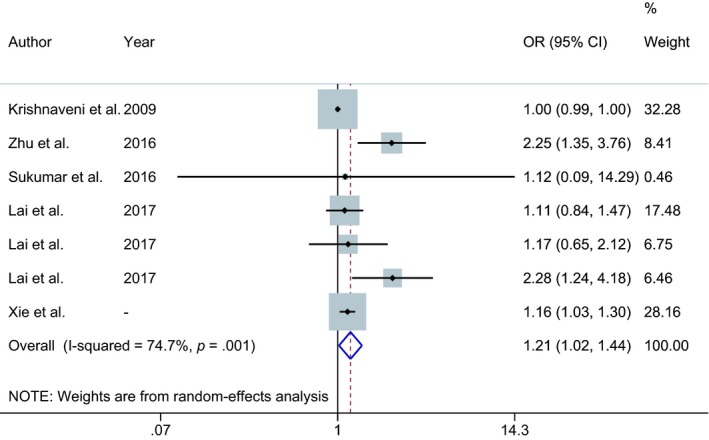
Forest plot showing the pooled effects of maternal folate status on the risk of GDM

Up to now, only a few studies explored the relationship between folate status (FA supplementation, plasma folate concentrations) and GMD risk. One cohort study in China has shown that daily FA supplement consumption was significantly associated with GDM risk (Zhu et al., 2016). In one recent study, Lai et al. (2018) observed that higher plasma folate concentrations were associated with higher odds of GDM. These results supported our findings that higher RBC folate concentrations were significantly associated with GDM risk. In our study, this significant association is still robust when the concentrations were assessed by different criteria (quintiles, proposed cutoff values, and log transformation). However, a few studies reported that serum folate concentrations were not associated with GDM risk (Krishnaveni et al., 2009; Sukumar et al., 2016). For example, Sukumar et al. (2016) reported no significant association between serum folate in the third trimester of pregnancy and GDM risk in a UK population. Several factors may account for the inconsistent findings. First, differences in measures of folate levels and critical time windows in pregnancy make comparisons challenging. Compared to the serum folate, the RBC folate indicates the average folate content of long‐lived red blood cells (Czeizel, Puho, Langmar, Acs, & Banhidy, 2010; Galloway & Rushworth, 2003; Pietrzik, Bailey, & Shane, 2010). Notably, exposure time is an important factor affecting the relation between levels of FA intake and adverse pregnancy outcomes (Wang et al., 2016). Second, given that the sample size in our study is relatively larger than that in previous studies, differences in power represent one possible explanation for the lack of association in earlier studies. Third, given the role of polymorphisms in folate status and GDM risk (Du et al., 2018; Hiraoka & Kagawa, 2017; Kanthimathi et al., 2017; Kwak et al., 2012), ethnic groups and dietary practices are indispensable factors for the conflicting results. To determine the effect of high folate on GDM risk, we conducted a mini meta‐analysis including four available studies and our study to determine the effect of folate on GDM risk (Table S1). As shown in Figure 1, there is a heterogeneity between the included studies (*I* ^2^ = 74.7%), and we observed a significantly increased risk of GDM in the random model (pooled OR = 1.21 (1.02, 1.44), *p* = .029). The findings further supported the association between high folate status and risk of GDM. Moreover, several studies have indicated that maternal high folate status was positively associated with insulin resistance in their offspring in human population studies and animal models (Huang et al., 2014; Yajnik et al., 2008). According to the Developmental Origins of Health and Disease (DOHaD) theory, which hypothesized that exposure to specific environmental factors in early life will determine the chronic diseases risk in future life, high folate status may have long‐term effects on insulin resistance.

The exact mechanism linking the effect of higher folate status and risk of GDM is still unclear. One possible explanation may be the interaction between excess folate intake and vitamin B12 metabolism (Paul & Selhub, 2017). Several studies have demonstrated that vitamin B12 deficiency worsened insulin resistance and was involved in the pathogenesis of GDM; high folate concentrations may further exaggerate the effect of vitamin B12 deficiency (Knight et al., 2015; Selhub, Morris, & Jacques, 2007). The other possible explanation is the harmful effects of unmetabolized folic acid (UMFA) in the blood, which could disturb cellular folate uptake and intracellular folate metabolism (Obeid & Herrmann, 2012). Furthermore, UMFA in blood is related to reduced natural killer (NK) cell cytotoxicity, which is involved in inflammation and insulin resistance, and thus participates in the pathogenesis of GDM (Bonamichi & Lee, 2017; Chiba et al., 2016; Zhao et al., 2011).

The major strength of this study was the availability of RBC folate levels during the second trimester of pregnancy in a Chinese population. Notably, previous studies have suggested that BMI before pregnancy and excessive weight gain in pregnancy are associated with the increased risk of GDM (Adane, Tooth, & Mishra, 2017; MacDonald, Bodnar, Himes, & Hutcheon, 2017). In the current study, we did not include these factors in our adjusted models, although maternal age, parity, and BMI at enrollment were adjusted. Therefore, we cannot completely exclude residual confounding. Moreover, because we do not have information on dietary and folic acid supplements, the association between folic acid supplements and GDM risk could not be further confirmed. Further studies with the combination of dietary intake, folic acid supplements, and blood biomarkers could strengthen the ability to detect the effect of folate on risk of GDM (Bailey et al., 2017). Additionally, we assessed the effect of RBC folate concentrations on GDM risk in one single center. Considering the geographical and ethnic differences in RBC folate concentrations (Hao et al., 2003; Marchetta & Hamner, 2016), further multicenter studies with large cohort populations should be performed to validate our study.

In conclusion, our study has provided epidemiological evidence that high RBC folate concentrations during the second trimester of pregnancy were significantly associated with GDM risk, indicating the need to evaluate high folate levels in pregnant women. Further longitudinal studies with diverse populations and different timing of folic acid supplementation are warranted to confirm our findings.

## CONFLICT OF INTEREST

The authors declare that they have no conflicts of interest.

## ETHICAL APPROVAL

All procedures performed in the study were in accordance with the ethical standards of the institutional research committee.
