# Supplementary material for: Association of maternal folate status in the second trimester of pregnancy with the risk of gestational diabetes mellitus
Source: Food Sci Nutr. 2019 Oct 18;7(11):3759–65. doi: 10.1002/fsn3.1235 (PMC6848811; doi:10.1002/fsn3.1235)
Supplement: Supplementary file 1 [file FSN3-7-3759-s001.doc]

| Supplementary Table 1. Information of folate status and GDM risk association studies included in the mini meta-analysis. | | | | | | | |
| --- | --- | --- | --- | --- | --- | --- | --- |
| Author [ref] | Year | Study design | Population | No. of cases/ controls | Methods of folate status assessment | Time of folate status assessment | OR/RR (95%CI) |
| Krishnaveni et al. | 2009 | Cross-sectional | Indian | 49/725 | Serum folate | 30 weeks’ gestation | 1.00 (0.99, 1.00) |
| Zhu et al. | 2016 | Prospective Cohort | Chinese | 249/1689 | FA supplement | First trimester | 2.25 (1.35, 3.76) |
| Sukumar et al. | 2016 | Case-control | UK | 143/201 | Serum folate | Third trimester | 1.12 (0.09, 14.29) |
| Lai et al. | 2017 | Cross-sectional | Chinese | 498^a^ | Plasma folate | 26-28 weeks’ gestation | 1.11 (0.84, 1.47) |
| Lai et al. | 2017 | Cross-sectional | Malay | 250^a^ | Plasma folate | 26-28 weeks’ gestation | 1.17 (0.65, 2.12) |
| Lai et al. | 2017 | Cross-sectional | Indian | 165^a^ | Plasma folate | 26-28 weeks’ gestation | 2.28 (1.24, 4.18) |
| Xie et al. [Our study] | - | Prospective Cohort | Chinese | 392/1890 | RBC folate | Second trimester | 1.16 (1.03, 1.30) |
| Abbreviation: UK, United Kingdom of Great Britain and Northern Ireland. FA, Folic acid. RBC, red blood cell. | | | | | | | |
| ^a^ The number is the total of cases and controls. | | | | | | | |
